# Systemic glucocorticoid therapy and adrenal insufficiency in adults: A systematic review

**DOI:** 10.1016/j.semarthrit.2016.03.001

**Published:** 2016-08

**Authors:** Rebecca M. Joseph, Ann Louise Hunter, David W. Ray, William G. Dixon

**Affiliations:** a NIHR Manchester Musculoskeletal Biomedical Research Unit, Central Manchester University Hospitals NHS Foundation Trust, Manchester Academic Health Science Centre, Manchester, UK; b Manchester Centre for Endocrinology and Diabetes, Institute of Human Development, The University of Manchester, Manchester Academic Health Science Centre, Manchester, UK; c Arthritis Research UK Centre for Epidemiology, Centre for Musculoskeletal Research, Institute of Inflammation and Repair, The University of Manchester, Manchester Academic Health Science Centre, Manchester, UK

**Keywords:** AI, adrenal insufficiency, IQR, inter-quartile range, GC, glucocorticoid, HPA-axis, hypothalamic–pituitary–adrenal axis, RCTs, randomised controlled trials, ACTH, adrenocorticotropic hormone, CRH, corticotropin-releasing hormone, IM, intra-muscular, IV, intravenous, HC, hydrocortisone, Glucocorticoids, Adrenal insufficiency, Systematic literature review

## Abstract

**Objectives:**

The aim of this systematic literature review was to summarize the current knowledge regarding the prevalence of, time to recovery from, and influence of glucocorticoid dose and duration on glucocorticoid-induced adrenal insufficiency (AI).

**Methods:**

Eligible studies were original research articles, which included adult patients with an indication for glucocorticoids and measured adrenal function following exposure to systemic glucocorticoids. Searches were performed in Web of Science and MEDLINE, with further articles identified from reference lists. Screening was performed in duplicate. Data were extracted for each group of glucocorticoid-exposed patients within eligible studies. The reported proportion of patients with AI was summarized as median and inter-quartile range. Results were then stratified by daily dose, cumulative dose, duration of exposure and time since last glucocorticoid use. The risk of bias within and across studies was considered: for randomised controlled trials risk of bias was assessed using the tool developed by the Cochrane Collaboration.

**Results:**

Overall, 73 eligible studies were identified out of 673 screened. The percentage of patients with AI ranged from 0% to 100% with a median (IQR) = 37.4% (13–63%). Studies were small—median (IQR) group size 16 (9–38)—and heterogeneous in methodology. AI persisted in 15% of patients retested 3 years after glucocorticoid withdrawal. Results remained widely distributed following stratification. AI was demonstrated at <5 mg prednisolone equivalent dose/day, <4 weeks of exposure, cumulative dose <0.5 g, and following tapered withdrawal.

**Conclusions:**

The heterogeneity of studies and variability in results make it difficult to answer the research questions with confidence based on the current literature. There is evidence of AI following low doses and short durations of glucocorticoids. Hence, clinicians should be vigilant for adrenal insufficiency at all degrees of glucocorticoid exposure.

## Introduction

Adrenal insufficiency (AI) is failure of the adrenal cortex to produce sufficient levels of cortisol. Chronically low cortisol levels can cause non-specific symptoms such as fatigue and nausea whilst lack of the usual cortisol response to stress can lead to a potentially fatal adrenal crisis [1]. Taking glucocorticoids (GCs) can lead to suppression of the hypothalamic–pituitary–adrenal (HPA) axis. The HPA-axis may remain suppressed following cessation of GC therapy, leaving the patient with adrenal insufficiency [2].

GCs are widely used therefore many patients could potentially be at risk of AI. In 2008, almost 1% of the UK adult population were exposed to oral GCs [3], including 0.79% on long-term courses (longer than 3 months) [4], and over 8 million prescriptions for oral GCs were issued in England during April 2014–March 2015 [5]. While there is limited published data around the clinical impact, one recent study suggests 6% of patients presenting at hospital with AI may have glucocorticoid-induced AI [6]. Looking forward, novel treatments aimed at improving the benefit:risk ratio of glucocorticoids (selective glucocorticoid receptor agonists, SEGRAs), which are currently being trialed, are unlikely to alter the risk of adrenal insufficiency. This is because AI is believed to result from the same mechanism that mediates the beneficial anti-inflammatory effects of glucocorticoids [7], [8].

AI has been a recognized side-effect of GC therapy since the early 1950s [9], [10], [11]; despite this, the risk of developing GC-induced AI remains unclear. Prevalence estimates from some of the larger observational studies range from 14% to 63% [12], [13], [14], [15]. It is also unclear whether or not the dose and duration of GC use affects the risk of developing AI: Schlaghecke et al. [12] found no relationship between either the dose or duration of therapy and AI, while Jamilloux et al. [13] found both cumulative dose and treatment duration to be associated with an increased risk of AI.

The aim of this systematic review was to summarize the published literature investigating GC-induced AI in adults. Specific objectives were to investigate (1) the prevalence of GC-induced adrenal insufficiency, (2) the time course of adrenal recovery following cessation of GC therapy and (3) the association between the dose and duration of GC therapy and the prevalence of adrenal insufficiency.

## Methods

### Eligibility criteria

Original research articles that tested adrenal function following exposure to glucocorticoids were identified. The population of interest were adult patients treated for the most common glucocorticoid indications as reported by van Staa et al. [16] for UK primary care data. These indications were disorders of the respiratory system; skin or subcutaneous tissue; musculoskeletal system or connective tissue; nervous system; digestive system; circulatory system; or neoplasms. Glucocorticoid use was limited to systemic routes including oral, intravenous, intramuscular, and subcutaneous. Finally, eligible papers were required to report the numbers of patients with normal/sub-normal HPA-axis function test results rather than presenting the average cortisol levels for study groups.

During data extraction, the following additional eligibility criteria were applied: papers were excluded if (1) participants aged under 16 were included; (2) glucocorticoids were taken less than 12 h before the HPA-axis function test; (3) glucocorticoid indications were unspecified; (4) the daily dose, duration and cumulative dose of GCs were all unreported; (5) it was clear that patients were using local GCs (topical, inhaled or intra-articular) during the study window; and (6) patients were pregnant or critically ill, were being treated with megestrol acetate, had metastatic cancer or were being treated peri-operatively (all potential confounders). Where it was possible to remove individual patients who met these exclusion criteria from the results, the paper was retained.

Published or in-press articles were included. There was no time restriction and non-English language articles were translated using Google Translate [17].

### Information sources

The search was performed in MEDLINE (1946-present) and Web of Science (1900-present). In addition, the reference lists of eligible papers were screened to identify further papers missed in the database search.

### Search strategy

The search included terms for adult humans, the indications described in Section Information sources, systemic glucocorticoids/named drug substances, and adrenal insufficiency/HPA-axis tests. The full search strategy is available in Supplementary File 1. Papers with only local glucocorticoids, in children or adolescents, or where the glucocorticoid was used as steroid cover/replacement therapy (e.g., in patients with Addison′s disease) were excluded. In the Web of Science search, the explicit mention of the HPA-axis, adrenal gland, or cortisol in the title of the article was required due to a large number of results in initial searches. The SIGN strategy search filters [18] were used to limit the MEDLINE results to clinical trials or observational studies. The Web of Science search was limited to journal articles, abstracts and proceedings. The latest search was performed on November 25, 2014.

### Study selection

The initial screening of search results by title and abstract was carried out in duplicate (R.J. and A.L.H.). Where there was disagreement, the article was discussed between the two reviewers and if there was still uncertainty the article was retained. Full texts were then assessed, in duplicate, for eligibility. Disagreements were resolved by discussion. The reference lists of eligible articles were then screened, and the eligibility of any articles identified was then checked in duplicate as before.

### Data extraction

Data were extracted using pre-designed forms. If a paper included multiple groups of GC-exposed patients, data were extracted for each group rather than pooling the data. For example, Suzuki et al. [19] compared patients exposed to 9 mg budesonide to patients exposed to 15 mg budesonide: data were extracted for each of these groups separately. Information extracted included the study design, number of participants, the age/gender distributions and indication, about comorbidities or other medications used; the GC indication; drug substance, route, dose, and duration; the type of HPA-axis test performed, and the criteria used to define AI, and the numbers of patients tested and the number of patients with AI.

### Summary measures

The data extracted are summarized in Table 1. The proportion of patients with AI in each group was extracted, and the data summarized using the median, inter-quartile range (IQR), and range. Summaries are presented for all groups and then stratified according to GC exposure, with dose converted to the prednisolone equivalent dose (Supplementary File 2). Groups were stratified by daily dose (<5 mg/day; 5–10 mg/day, 10–20 mg/day; 20+ mg/day), duration (<4weeks, 4–52weeks, 52+weeks) and cumulative dose (<0.5 g, 0.5–5 g, 5+ g). To investigate the time to adrenal recovery, groups were further stratified according to the timing of the test with respect to the last dose of GCs, limited to groups exposed to oral GCs only. These results were summarized as for the dose/duration; in addition, summaries of individual studies that tested patients multiple times after cessation of GCs are presented. Studies that used a tapered dose of GCs are also summarized individually.

### Risk of bias/study quality

The Cochrane Collaboration′s tool for assessing the risk of bias [20] was applied to the randomised controlled trials (RCTs) included in the review, including an additional section to assess the risk of confounding in these studies. The quality of reporting and heterogeneity of the other included studies, and the potential for bias across studies, are commented on in the results and discussion.

## Results

**Fig. 1:**
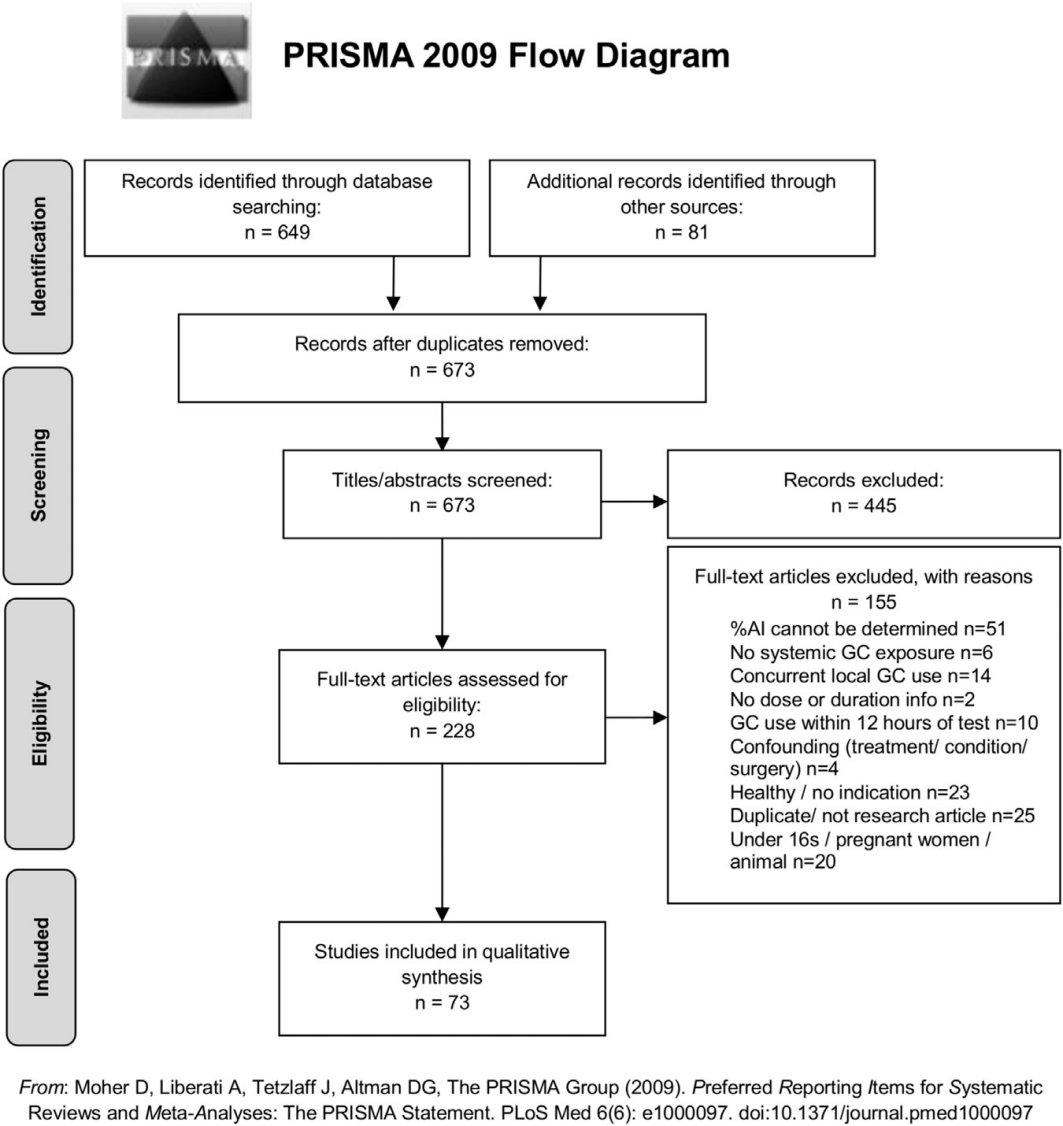
Flow diagram showing the study selection process. Based on the PRISMA 2009 flow diagram [21]. Abbreviations: AI, adrenal insufficiency; GC, glucocorticoid.

In total, 673 articles were screened and 73 studies were included in the final review (Fig. 1). Within the 73 studies there were 100 groups of GC-exposed patients and a total of 3166 patients. A summary of these groups is presented in Table 1, while a condensed version of the data extraction table can be seen in Supplementary File 3. There were thirteen RCTs [19], [22], [23], [24], [25], [26], [27], [28], [29], [30], [31], [32], [33] with random allocation and blinding maintained throughout; the remaining 60 studies were classified as observational studies [12], [13], [14], [15], [34], [35], [36], [37], [38], [39], [40], [41], [42], [43], [44], [45], [46], [47], [48], [49], [50], [51], [52], [53], [54], [55], [56], [57], [58], [59], [60], [61], [62], [63], [64], [65], [66], [67], [68], [69], [70], [71], [72], [73], [74], [75], [76], [77], [78], [79], [80], [81], [82], [83], [84] with some open trials [85], [86], [87], [88] and pilot studies [89]. The majority of groups were small (median = 16 patients). The most frequent GC indications were musculoskeletal, respiratory, and digestive tract conditions and the most common GCs investigated were prednisolone, prednisone, and budesonide. The majority of studies investigated oral GCs.

Across all of the groups the median percentage of patients found to have adrenal insufficiency was 37.4% (IQR: 13–63%), and this was similar for RCTs (median = 37.4%, IQR: 11–60%) and observational studies (median = 37.2%, IQR: 13–63%). Results were widely distributed in each category, with a range of 0–92% for RCTs and 0–100% for observational studies.

**Fig. 2:**
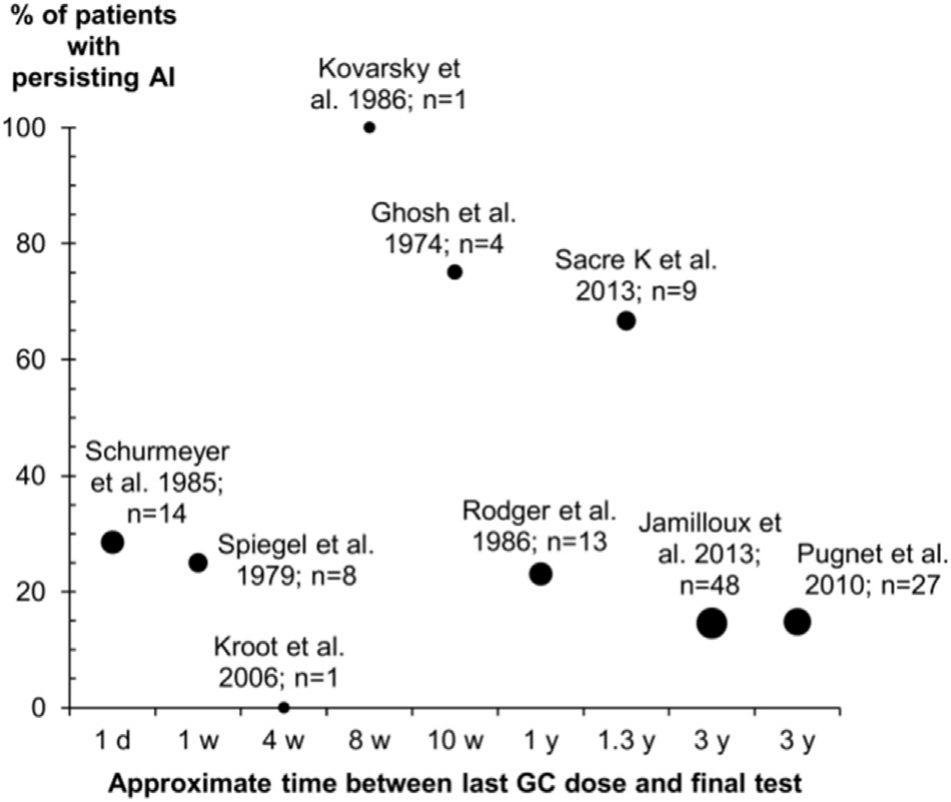
Adrenal recovery over time. Abbreviations: AI adrenal insufficiency; GC, glucocorticoid; d, day; w, week; y, year. Each point represents a study in which HPA-axis tests were repeated at least once following withdrawal of GC therapy (in all patients, in patients with AI at the initial test, or in a subset of the latter). Horizontal axis: time between the final GC dose and the final test performed during the study. Vertical axis: percentage of patients found to have AI at the final test. Points are labeled with the total number of patients retested during the studies, including those who did and did not recover by the end of follow-up. The size of the point reflects the number of patients retested.

Table 2 shows the proportion of patients with AI for those taking oral GCs stratified by the timing of the test with respect to their most recent dose. Some patients were found to have adrenal insufficiency when tested more than 30 days after their last oral GC dose. In nine studies, patients were retested several times after GC withdrawal (Fig. 2). The number of patients retested ranged from 1 [61], [89] to 48 [13]. Excluding the studies retesting a single patient, the percentage of patients with AI persisting at the end of follow-up ranged from 75% (after 10 weeks of cessation) [48] to 15% (after 3 years of cessation) [13], [73]. Four studies followed patients for at least 1 year and in these studies the percentage of patients with persisting AI ranged from 15% to 67% [13], [14], [73], [75]. It was not always clear which patients had been selected for follow-up and two studies lost a large proportion of patients to follow-up (35–40%) [13], [73].

There was no obvious pattern when stratifying by the dose, duration or cumulative dose (Table 3). Across all strata, the median percentage of patients with adrenal insufficiency ranged from 14% (IQR: 0–40%) for a medium cumulative dose (0.5–5 g) to 50% (IQR: 35–66%) for a high cumulative dose (greater than 5 g).

A total of 13 studies tested adrenal function after a GC taper and these are summarized in Table 4. The median percentage of patients with AI after a GC taper was 38% and ranged from 0% to 84%. The studies varied in the initial GC doses, duration of tapering and prior exposure pattern.

The risk of bias assessment for RCTs is presented in Supplementary File 4. Where reported, studies scored a low risk in most domains. In many cases, however, insufficient information was provided on which to base a judgement. The risk of attrition bias scored poorly: three out of the 13 RCTs reported outcomes for all tested patients; in the remaining papers it was either impossible to calculate the number of patients tested or the outcome was missing for some patients with no explanations. The risk of confounding was high for many of the studies: in seven of the studies, some patients demonstrated abnormal HPA-axis function at baseline.

The risk of confounding is likely to be high in the observational studies: there was no information at all about concurrent medical conditions for 56 of the 76 groups, and no information about concurrent treatments in 46 groups. The exclusion of any local GC use was explicitly mentioned for 17 of the groups and therefore assumed in the remainder. The heterogeneity of these studies can be seen in Table 1, which shows the range of indications, drug substances, routes and adrenal function tests used. In general very few details were provided regarding patient selection, and loss to follow-up was difficult to ascertain.

## Discussion

Across the 100 GC-exposed participant groups included in this review, the median prevalence of AI was 37%. Whilst this figure is in line with the estimates of the larger observational studies (14–63% [12], [13], [14], [15], it is unlikely to be a meaningful prevalence estimate given the wide distribution of our results and resultant imprecision. Most studies retesting patients after GC withdrawal found evidence of persisting AI, including 15% of patients retested after 3 years of cessation (Fig. 2), yet from the current literature it is not possible to describe a time course for adrenal recovery. There is also evidence that AI may persist for more than 3 years after GC withdrawal. When results were stratified according to the average dose, duration and cumulative dose of GCs used within groups, no clear trends were revealed. Within the stratifications, results were still widely distributed and AI was demonstrated even in the lowest exposure categories. AI was also found in studies which included a tapered reduction in dose.

It has not been possible to address any of our research questions adequately, based on over sixty years of published literature. There are several factors that would contribute to the wide distribution of the results. Firstly, the studies found were heterogeneous, including variation in indications and GC type, dose, and duration. There was also variability in methods of outcome assessment: while the short ACTH test (standard or low dose) was the most frequently used, a range of other tests including the insulin tolerance test (the gold-standard), ACTH infusion and CRH test were also used. Even within the same test, the cutoff values used to define AI varied from study to study. This heterogeneity in study methods is likely to increase the variability in the results and make it difficult to appropriately summarize the findings. Nonetheless, stratification led to no greater clarity in the results. Secondly, the group sizes included were small—50% had fewer than 16 patients—and so the individual prevalence estimates for each group are less precise. Another factor is having only group-level exposure data; for the majority of studies the dose and duration of GC use within the groups were variable and basing the stratification on average values may be one reason no obvious trends were apparent.

In addition to these, it is possible that bias was present within and across studies, although in most cases there was not enough evidence to be able to comment on the risk of bias. Only 6 of the 62 observational studies were published after the publication of the STROBE statement [90] in 2007 and few details were provided in any of the observational studies regarding the selection and recruitment of patients, how GC exposure was assessed, any loss to follow-up, or any comorbidities or therapies that may have confounded the results. While the majority of the RCTs were published after the publication of the CONSORT statement [91], many still lacked sufficient detail in some of the domains. This included incomplete outcome reporting making it difficult again to judge the risk of attrition bias for most studies. Importantly, many of the RCTs reported evidence of adrenal insufficiency prior to exposure to the study drug making it difficult to attribute AI to the effects of the study drug. There is also potential for bias across studies, although plotting the % AI against the group size did not reveal any obvious gaps (Supplementary File 5). It is possible that some patient groups are underrepresented in the literature: van Staa et al. [16] demonstrated that the most frequently recorded indication for oral GCs was respiratory disease (40% of GC users), yet 24% of the papers focused on respiratory disease, compared to 25% for musculoskeletal and 19% for gastro-intestinal conditions (Table 1).

While this manuscript was in preparation, Broersen et al. [92] published a systematic review on the same subject, with similar research questions and methods. The authors used a logistic regression model to generate pooled estimates of the percentage of patients with adrenal insufficiency; their overall result for patients using oral GCs was 48.7% (95% CI: 36.9–60.6). We decided at study inception, before our final search and data extraction, not to perform a meta-analysis, based on the heterogeneity shown in a few key references. Broersen et al. examined the association with GC dose and duration in asthma patients only, to create a more homogeneous population. Their results suggest an increased risk of AI associated with increased dose and duration of GCs, although confidence intervals overlapped. No such pattern was seen within our whole study population.

A strength of our study is the rigorous methodology followed, in duplicate, to identify suitable studies and extract data. It is reassuring that, despite slight differences in search strategy, including restricting our database searches to certain GC indications, the included studies overlap almost completely with those of another, independent, group [92]. This suggests that our search strategy was effective. We did not restrict results to English-language papers or a minimum study size, therefore we have included some additional papers compared to the study of Broersen et al. A possible weakness is the fact we tightened the eligibility criteria during data extraction, as described in the methods. The reason for this was the lack of detail and ambiguity in many of the articles, which became increasingly apparent when looking at papers in increasing detail and led us to write out a strict list of inclusion/exclusion criteria for this stage. We did not perform a risk of bias assessment for the observational studies because the quality of reporting was poor in the majority of studies, therefore, it would not have been possible to give a judgement of high/low risk in many cases.

From our results, it is clear that research is still needed into the risk of AI following GC exposure. Future studies should aim to have a sufficient sample size to estimate the prevalence of adrenal insufficiency with confidence. Studies could include patients who have been withdrawn from GCs as there remains a lack of published data regarding how long adrenal insufficiency persists. There is also a lack of published research regarding differing tapering schedules, a question potentially suited to a randomised controlled trial design. Finally, accurate patient-level data regarding GC exposure is important if the dose and duration is to be studied: ideally studies should include long-term prospectively collected data. Alongside these considerations, it is vital that quality of reporting is increased so that the generalizability, risk of bias and potential confounding can be assessed. Use of available guidelines, such as the STROBE [90] or CONSORT [91] statements, should ensure all important items are reported in future articles.

Our study raises questions for clinicians prescribing GC therapy. The major endocrinology societies (Endocrine Society [United States], Society for Endocrinology [United Kingdom], European Society of Endocrinology) do not currently issue clinical guidance on reducing GC-induced AI. UK clinicians can turn to the British National Formulary [93] and the National Institute for Health and Care Excellence Clinical Knowledge Summary [94], which both advise gradual GC withdrawal if patients have taken: more than 40 mg prednisolone (or equivalent) daily for more than one week; repeated GC doses in the evening; GCs for more than three weeks; a short course of GCs within one year of stopping long-term GC therapy; or have other risk factors for adrenal suppression. With regards to tapering, they advise rapid reduction to a physiological GC dose (7.5 mg prednisolone daily or equivalent), and slow reduction thereafter. However, our study suggests that the evidence base supporting these recommendations is not robust. Furthermore, we highlight that studies demonstrate AI at all levels of GC exposure, even low dose and after tapering. We therefore suggest that clinicians be vigilant for GC-induced AI with all degrees of GC exposure, and counsel patients accordingly. Anecdotally, testing for adrenal insufficiency in patients with chronic GC exposure is infrequent. Adrenal function can be tested using the short ACTH stimulation test (250 µg), as performed in 57% groups in this study; this produces equivalent results to the gold-standard insulin tolerance test (used in 14%), but is safer, and carries good long-term predictive value [95], [96]. Regarding who should be tested, and when, there is an imperative need for high quality, prospective studies to better guide clinicians, and reduce patient morbidity.

## Contributorship statement

All authors participated in the design of the study. RJ and WD developed the search strategy. RJ and ALH performed screening, data extraction and analysis, and drafted the manuscript. All authors read and approved the final article.

## Figures and Tables

**Table 1:** Summary characteristics of the groups included in the review.

Characteristics by group	Overall (*n* = 100)	Study design	Observational (*n* = 76)
		RCT (*n* = 24)	
Total number of patients tested	3166	795	2371
Median (IQR) number of patients tested	16 (9–38)	25 (17–48)	14.5 (8–36)
Range number of patients tested	2–399	7–86	2–399
Median (IQR) average age*	45 (36–53)	35.5 (33–37)	48 (41–54)
Median (IQR) % female*	51 (40–67)	58.5 (32–67)	50 (40–67)

**Glucocorticoid indication**			
Musculoskeletal	25	3	22
Respiratory	24	7	17
Neoplasms	4	0	4
Digestive system	19	14	5
Nervous system	3	0	3
Transplant	7	0	7
Multiple	18	0	18

**Drug substances**			
Prednisone	17	4	13
Prednisolone	24	6	18
Budesonide	16	14	2
Methylprednisolone	8	0	8
Triamcinolone	5	0	5
Dexamethasone	4	0	4
Other (hydrocortisone, paramethasone, fluocortolone)	4	0	4
Unclear	5	0	5
Multiple	17	0	17

**Glucocorticoid Route**			
Oral	87	24	63
IM	5	0	5
IV	3	0	3
Multiple	5	0	5
Dose category**			
<5 mg/day	15	8	7
5–20 mg/day	42	13	29
20+ mg/day	23	3	20
Unknown	20	0	20

**Duration category**			
<4 weeks	15	4	11
4–52 weeks	36	20	16
52+ weeks	37	0	37
Unknown	12	0	12

Cumulative dose category**			
<0.5 g	27	19	8
0.5–5 g	24	5	19
5+ g	13	0	13
Unknown	36	0	36

**Adrenal function test**			
Short ACTH test	57	17	40
Insulin tolerance test	14	0	14
ACTH infusion	8	5	3
CRH test	6	0	6
Metyrapone test	4	0	4
Plasma cortisol	8	2	6
Urinary cortisol	2	0	2
Multiple	1	0	1

RCT, randomised controlled trial; IQR, inter-quartile range; IM, intra-muscular; IV, intravenous; ACTH, adrenocorticotropic hormone; CRH, corticotropin-releasing hormone.

Unless otherwise indicated, figures represent the number of studies with that characteristic.

⁎ Results may be for whole group not necessarily those tested. May be pooled for the whole study. 17/100 groups missing age; 15/100 groups missing gender.

⁎⁎ Prednisolone equivalent dose.

**Table 2:** Percentage of patients per group with AI by time since last dose (oral glucocorticoids only).

Time since last dose	Total number of patients	Number of groups	Median (range) group size	Median (IQR), % AI	Range, % AI
Up to 1 day	2542	63	21 (3–399)	40.9 (20–63)	0–100
2–6 days	323	20	10 (2–75)	35.9 (0–71)	0–90
7–29 days	49	2	24.5 (4–45)	16.7 (0–33)	0–33
30+ days	31	2	15.5 (15–16)	47.9 (27–69)	27–69

IQR, inter-quartile range; AI adrenal insufficiency.

**Table 3:** Percentage of patients per group with AI by glucocorticoid dose, duration or cumulative dose.

	Total number of patients	Number of groups	Median (range) group size	Median (IQR), % AI	Range, % AI
Average daily dose*					
<5 mg/day	371	15	21 (6–63)	22.7 (11–36)	0–62
5–10 mg/day	703	22	22 (7–86)	43.7 (38–58)	14–80
10–20 mg/day	623	16	19 (3–279)	33.3 (22–80)	0–100
20+ mg/day	527	26	8 (2–100)	16.3 (0–71)	0–100

**Duration**					
<4weeks	378	15	9 (4–86)	36.4% (0–89%)	0–100%
4–52 weeks	1533	36	20 (5–399)	33.9% (12–55%)	0–92%
52+ weeks	1093	37	19 (3–150)	42% (26–65%)	0–100%
Cumulative dose*					
<0.5g	702	28	19 (2–86)	35.4% (11–54%)	0–100%
0.5–5g	804	23	10 (4–279)	14% (0–40%)	0–89%
5+ g	491	13	23 (3–150)	50% (35–66%)	0–100%

IQR inter-quartile range; AI adrenal insufficiency

⁎ Prednisolone equivalent dose.

**Table 4:** Summary of studies testing adrenal function after tapering GCs.

References	Initial dose* (mg)	Final dose* (mg)	Duration of tapering	Rate of tapering	HC or ACTH cover?	Overall average daily dose* (mg)	Overall duration (weeks)	Overall total dose* (g)	*N* tested	% AI
Cydulka and Emerman [24]	40	5	8 days	5 mg/day	None	22.5	1.1	0.18	8	0
Miro et al. [70]	Mean = 62	0	Mean = 30 days	10 mg/3days then 2.5 mg/3days	None	NS	5	3.9	8	12.5
Miro et al. [70]	Mean = 61	0	Mean = 30 days	10 mg/3days then 2.5 mg/3days	None	NS	4.3	0.78	14	14.3
Barrier et al. [36]	7	0	NS	1 mg/?	All	7	NS	NS	22	31.8
Boots et al. [39]	20	10	4 weeks	5 mg/2 weeks	None	10	11	1.53	42	33.3
Bacon et al. [35]	NS	4–10	NS	1 mg/month	Some	NS	393	24.8	23	34.8
Havranek et al. [88]	50	10	6 days	8 mg/day	None	7.5	17	0.87	8	38
Pugnet et al. [73]	Mean = 65.5	5	NS	NS	None	65.5	142	NS	100	45
Jamilloux et al. [13]	Mean = 51	5	~34 weeks	10 mg/2 weeks, 5 mg/2 weeks, 2.5 mg/2 weeks, 1 mg/month	None	NS	74.1	7.7	150	49.3
Rutgeerts et al. [31]	40	5	10 weeks	3.5 mg/week	None	24	10	1.68	NS: <86	53
Schlaghecke et al. [77]	60	20	4 weeks	10 mg/week	None	45	4	12.6	9	55.6
Desrame et al. [44]	NS	4–7.5	NS	10 mg/10 days to 0.5 mg/7days	Some	5.5	34	6.7	55	65.5
Rodger et al. [75]	7.5	2.5	NS	1.2–2.5 mg/month	Some	NS	248	25.9	19	68.4
Campieri et al. [23]	40	5	9 weeks	4 mg/week	None	40	8.6	1.4	58	84

HC, hydrocortisone; ACTH, adrenocorticotropic hormone/corticotropin; *N*, number; AI, adrenal insufficiency.

⁎ Prednisolone equivalent dose.
