# Supplementary material for: Systemic glucocorticoid therapy and adrenal insufficiency in adults: A systematic review
Source: Semin Arthritis Rheum. 2016 Aug;46(1):133–41. doi: 10.1016/j.semarthrit.2016.03.001 (PMC4987145; doi:10.1016/j.semarthrit.2016.03.001)
Supplement: Supplementary file 1 — Supplementary material [file mmc1.pdf]

Supplementary File 1 Full search strategy

Search strategy: last run 25.11.2014

Medline

Ran on 25.11.2014 with 858 results prior to adding in paper type limits, 285 after

1. exp human/
2. exp neoplasms/
3. exp musculoskeletal diseases/
4. exp digestive system diseases/
5. exp respiratory tract diseases/
6. exp nervous system diseases/
7. exp cardiovascular diseases/
8. exp "skin and connective tissue diseases"/
9. (respiratory or inflamm*).mp.
10. or/2-9
11. ((glucocorticoid? or glucocorticosteroid? or steroid? or corticosteroid? or cortisone or hydrocortisone) adj3 (systemic or therapy or treat* or oral or tablet? or inject* or intravenous or intra*muscular or exogenous)).mp.
12. (cortin or methylprednis* or predniso* or b*met?asone or triamcinolone or budesonide).mp.
13. 11 or 12
14. adrenal insufficiency/
15. exp addison disease/
16. ((hypothalam* or pituitary or hypophys* or adrenal or HPA or adrenocort*) adj5 (insufficien* or suppres* or inhibit* or hypofunction or damage? or alter* or integrity)).mp.
17. ((synacthen or tetracoside or tetracosactin or ACTH or "adrenocorticotropic hormone" or cosyntropin or metyrapone or metopirone or metapyrone or CRH or "insulin tolerance" or "insulin stress") adj2 (stimulat* or test)).mp.
18. ("baseline cortisol" or "cortisol level").mp.
19. or/14-18
20. 1 and 10 and 13 and 19
21. (intra$articular or intravitreal or intranasal or epidural or inhale* or aerosol or topical or cream or ointment or ((knee or hip or shoulder) and inject*)).mp.
22. ((oral or tablet* or intravenous or intra$muscular).mp. or systemic.ti.)
23. 21 not 22
24. (exp adolescent/ or exp child/ or exp infant/) not exp adult/
25. (infant* or neonat* or child*).ti.
26. (addison* or hyp*plasia or "primary adrenal" or "replacement therapy").ti.
27. or/23-26
28. 20 not 27
29. 28 not "Review".sa_pubt.

Limit to clinical trials or observational studies using the SIGN strategy's filters(1)

SIGN filters:
1. Randomized Controlled Trials as Topic/
2. randomized controlled trial/
3. Random Allocation/
4. Double Blind Method/
5. Single Blind Method/
6. clinical trial/
7. clinical trial, phase i.pt.
8. clinical trial, phase ii.pt.
9. clinical trial, phase iii.pt.
10. clinical trial, phase iv.pt.
11. controlled clinical trial.pt.
12. randomized controlled trial.pt.
13. multicenter study.pt.
14. clinical trial.pt.
15. exp Clinical Trials as topic/
16. 1 or 2 or 3 or 4 or 5 or 6 or 7 or 8 or 9 or 10 or 11 or 12 or 13 or 14 or 15
17. (clinical adj trial$).tw.
18. ((singl$ or doubl$ or treb$ or tripl$) adj (blind$3 or mask$3)).tw.
19. PLACEBOS/
20. placebo$.tw.
21. randomly allocated.tw.
22. (allocated adj2 random$).tw.
23. 17 or 18 or 19 or 20 or 21 or 22
24. 16 or 23
25. case report.tw.
26. letter/
27. historical article/
28. 25 or 26 or 27
29. 24 not 28
30. Epidemiologic studies/
31. exp case control studies/
32. exp cohort studies/
33. Case control.tw.
34. (cohort adj (study or studies)).tw.
35. Cohort analy$.tw.
36. (Follow up adj (study or studies)).tw.
37. (observational adj (study or studies)).tw.
38. Longitudinal.tw.
39. Retrospective.tw.
40. Cross sectional.tw.
41. Cross-sectional studies/

42. or/30-41
43. 29 or 42

Web of Science 364 results

1. TS=(humans or patients or participants or volunteers or adults or women)
2. ts=inflamm* OR ts=(rheumat* or arthr* or lupus or musculoskeletal or "connective tissue") OR ts=(respiratory or bronch* or asthma or COPD or pulmonary or "cystic fibrosis") OR ts=(digestive or gastr* or intestin* or bowel or enteritis or colitis or chron*s) OR ts=(skin or eczema or uticaria or psoriasis or cutaneous or sub*cutaneous or dermatol*) OR ts=(neoplasm* or cancer) OR ts=(arteries or circulatory or vasculitis or arteritis) OR ts=(nervous or nerve or eye or ocular or ophthal*)
3. TS=((glucocorticoid$ or glucocorticosteroid$ or steroid$ or corticosteroid$ or cortisone or hydrocortisone) NEAR/3 (systemic or therapy or treat* or oral or tablet$ or inject* or intravenous or exogenous))
4. TS=(cortin or methylprednis* or predniso* or b*met$asone or triamcinolone or budesonide)
5. #4 OR #3
6. TS=((hypothalam* or pituitary or hypophys* or adrenal or HPA or adrenocort*) NEAR/5 (insufficien* or suppres* or inhibit* or hypofunction or damage$ or alter* or integrity))
7. TS=((synacthen or tetracoside or tetracosactin or ACTH or "adrenocorticotropic hormone" or cosyntropin or metyrapone or metopirone or metapyrone or CRH or "insulin tolerance" or "insulin stress") NEAR/2 (stimulat* or test))
8. TS=("baseline cortisol" or "baseline cortisol" or "plasma cortisol" or "serum cortisol")
9. #8 OR #7 OR #6
10. #9 AND #5 AND #2 AND #1
11. TI=((glucocorticoid$ or glucocorticosteroid$ or steroid$ or corticosteroid$) NEAR/3 (therapy or treat* or oral or tablet$ or inject* or intravenous or exogenous))
12. TI=(hydrocortisone or cortisone or cortin or methylprednis* or predniso* or b*met$asone or triamcinolone or budesonide)
13. #12 or #11
14. TI=((hypothalam* or pituitary or hypophys* or adrenal or HPA or adrenocort*) NEAR/5 (insufficien* or suppres* or inhibit* or hypofunction or damage$ or alter* or integrity))
15. TI=((synacthen or tetracoside or tetracosactin or ACTH or "adrenocorticotropic hormone" or cosyntropin or metyrapone or metopirone or metapyrone or CRH or "insulin tolerance" or "insulin stress") NEAR/2 (stimulat* or test))
16. TI=("baseline cortisol" or "plasma cortisol" or "serum cortisol")
17. #16 OR #15 OR #14
18. #17 AND #13
19. #18 OR #10
20. TI=(adrenal or adrenocortical or HPA or pituitary or hypophysis or hypothalam* or cortisol)
21. #20 AND #19

22. TS=(intra*articular or intravitreal or intranasal or epidural or inhale* or topical or cream or ointment or aerosol or ((knee or hip or shoulder) and inject*)) not (ts=(oral or tablet* or intravenous or intra$muscular) or ti=systemic)
23. TS=(infant* or child* or neonat* or newborn*)
24. TI=(guinea or rat or monkey or horse* or dog* or canine or feline or mice or mouse or trout)
25. TI=(addison* or "primary adrenal" or replacement or *adenoma or adrenoleukodystrophy or hyp*plasia)
26. #25 OR #24 OR #23 OR #22
27. #21 not #26

AND DOCUMENT TYPES: (Article OR Abstract of Published Item OR Meeting Abstract OR Meeting Summary OR Proceedings Paper)

References

1. Scottish Intercollegiate Guidelines Network. Search Filters. 2015 [cited 03/09/2015]; Available from: http://www.sign.ac.uk/methodology/filters.html
