# Supplementary material for: Systemic glucocorticoid therapy and adrenal insufficiency in adults: A systematic review
Source: Semin Arthritis Rheum. 2016 Aug;46(1):133–41. doi: 10.1016/j.semarthrit.2016.03.001 (PMC4987145; doi:10.1016/j.semarthrit.2016.03.001)
Supplement: Supplementary file 2 — Supplementary material [file mmc2.pdf]

Supplementary File 2 Prednisolone equivalent dose

The following figures were used to convert glucocorticoid doses into prednisolone equivalent doses. In the case of budesonide, an estimate of the potential to suppress plasma cortisol levels was used as this was deemed more relevant than the therapeutic equivalence.

5mg Prednisolone is equivalent to[1-3]:

Prednisone	5mg
Hydrocortisone	20mg
Dexamethasone	750µg
Methylprednisolone	4mg
Triamcinolone	4mg
Fluocortolone	5mg
Paramethasone	2mg

20mg Prednisolone is equivalent to[4]:

Budesonide	29mg

1. Joint Formulary Committee. Glucocorticoid therapy. *British National Formulary*. 65 ed. London: BMJ Group and Pharmaceutical Press, 2013:462.
2. Beris P, Burger A, Favre L, Riondel A, Miescher PA. Adrenocortical responsiveness after discontinuous corticosteroid therapy. *Klinische Wochenschrift* 1986;64(2):70-5.
3. Hu C. Steroid Equivalence Converter, 1999.
4. Edsbacker S, Nilsson M, Larsson P. A cortisol suppression dose-response comparison of budesonide in controlled ileal release capsules with prednisolone. *Aliment Pharmacol Ther* 1999;13(2):219-24.
