# Supplementary material for: Systemic glucocorticoid therapy and adrenal insufficiency in adults: A systematic review
Source: Semin Arthritis Rheum. 2016 Aug;46(1):133–41. doi: 10.1016/j.semarthrit.2016.03.001 (PMC4987145; doi:10.1016/j.semarthrit.2016.03.001)
Supplement: Supplementary file 4 — Supplementary material [file mmc4.pdf]

Supplementary File 4 – risk of bias in RCTs

Risk of bias in the included randomised controlled trials, assessed using the Cochrane Collaboration's tool for assessing risk of bias:

Chapter 8: Assessing risk of bias in included studies. In: Higgins JPT, Green S (editors). *Cochrane Handbook for Systematic Reviews of Interventions* Version 5.1.0 [updated March 2011]. The Cochrane Collaboration, 2011. Available from www.cochrane-handbook.org.

Papers assessed:

1. Aaronson et al. 1998
2. Campieri et al. 1997
3. Cydulka et al. 1998
4. Feiss et al. 1992
5. Ferguson et al. 1998
6. Greenberg et al. 1996
7. Hellers et al. 1999
8. Kirwan et al. 2006
9. Lofberg et al. 1996
10. Rutgeerts et al. 1994
11. Sorkness et al. 1999
12. Suzuki et al. 2013
13. Vargas et al. 1998

Concerned with whether any methodological factors could influence the prevalence of AI found in any of the groups. Part 7 of the tables is based on potential for confounding: prior/concurrent steroid therapy, other conditions and other therapies. (Yes, No, Unreported).

Table 1 Risk of bias summary, by paper

	1) Random sequence generation (selection bias)	2) Allocation concealment (selection bias)	3) Blinding of participants and personnel (performance bias)	4) Blinding of outcome assessment (reporting bias)	5) Incomplete outcome reporting (attrition bias)	6) Selective reporting (reporting bias)	7) Risk of confounding (prior exposure; other drugs/conditions; reporting of other steroids; baseline adrenal function)
Aaronson et al. 1998	?	?	+	+	+	?	+
Campieri et al. 1997	?	?	+	+	?	?	-
Cydulka et al. 1998	+	?	+	+	+	?	?
Feiss et al. 1992	?	?	+	+	+	?	+
Ferguson et al. 1998	+	+	+	+	?	?	-
Greenberg et al. 1996	+	?	+	+	?	?	-
Hellers et al. 1999	?	+	+	+	?	?	-
Kirwan et al. 2006	+	+	+	+	?	?	-
Lofberg et al. 1996	+	?	+	+	-	?	-
Rutgeerts et al. 1994	+	?	+	+	?	?	?
Sorkness et al. 1999	+	?	+	+	-	?	+
Suzuki et al. 2013	+	?	+	+	-	?	-
Vargas et al. 1998	?	?	+	+	?	?	+

Low risk	+
Unclear risk	?
High risk	-

Figure 1 Risk of bias graph

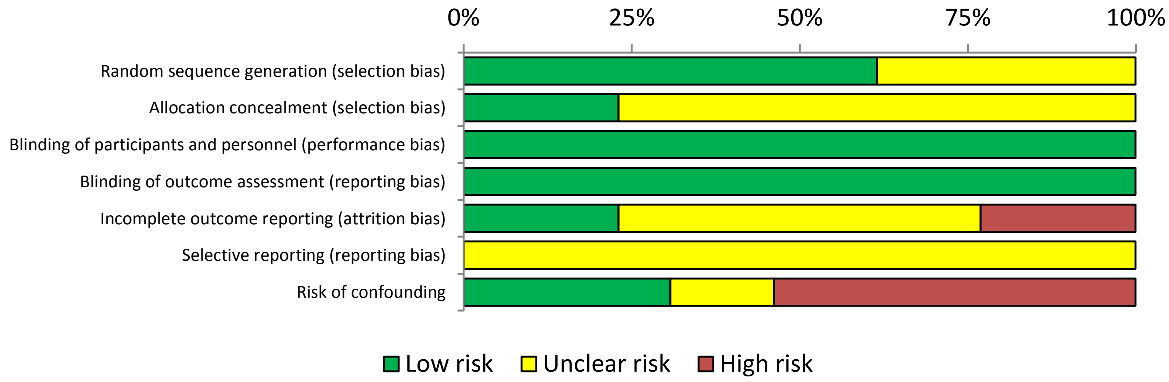

1. Aaronson et al. 1998

Paper assessed:

Aaronson D, Kaiser H, Dockhorn R, Findlay S, Korenblat P, Thorsson L, et al. Effects of budesonide by means of the Turbuhaler on the hypothalmic-pituitary-adrenal axis in asthmatic subjects: a dose-response study. The Journal of Allergy and Alinical Immunology 1998;101(3):312-9.

Entry	Judgement	Support for judgement
1) Random sequence generation (selection bias)	Unclear risk	Comment: not discussed
2) Allocation concealment (selection bias)	Unclear risk	Comment: not discussed
3) Blinding of participants and personnel (performance bias)	Low risk	P313: "To maintain blinding, patients assigned to the budesonide groups or the inhaled placebo group received placebo prednisone capsules; patients allocated to the oral prednisone group were issued inhaler devices containing placebo."
4) Blinding of outcome assessment (reporting bias)	Low risk	Comment: not discussed; ACTH test unlikely to be influenced by knowledge of allocation.
5) Incomplete outcome reporting (attrition bias)	Low risk	Comment: 13 patients randomised to oral prednisolone. One patient withdrawn due to "poor compliance or failure to fulfil inclusion criteria" (p315).
6) Selective reporting (reporting bias)	Unclear risk	Comment: no link or reference to protocol.
7) Risk of confounding (prior exposure; other drugs/conditions; reporting of other steroids; baseline adrenal function)	Low risk	Comment: excluded patients with recent hospitalization due to asthma, recent respiratory tract infection or history of significant illness. Patients had no corticosteroids of any kind for 6 months. Further, patients with abnormal basal plasma cortisol were excluded.

2. Campieri et al. 1997

Paper assessed:

Campieri M, Ferguson A, Doe W, Persson T, Nilsson LG. Oral budesonide is as effective as oral prednisolone in active Crohn's disease. The Global Budesonide Study Group. Gut 1997;41(2):209-14.

**Entry**	**Judgement**	**Support for judgement**
1) Random sequence generation (selection bias)	Unclear risk	P210 "The patients were subsequently randomised to treatment with either…"<br><br>Comment: no further details given.
2) Allocation concealment (selection bias)	Unclear risk	Comment: no details given.
3) Blinding of participants and personnel (performance bias)	Low risk	P210: "randomised double blind, double dummy study".<br>P210: "drugs were provided in identical blister packages".<br><br>Comment: assume done.
4) Blinding of outcome assessment (reporting bias)	Low risk	Comment: unclear whether this was blind; ACTH test unlikely to be influenced by knowledge of allocation.
5) Incomplete outcome reporting (attrition bias)	Unclear risk	Comment: report only % (Table2) so is not possible to see the numbers after treatment; unclear if all were tested. 36/177 discontinued treatment but cannot tell which groups these were in.
6) Selective reporting (reporting bias)	Unclear risk	Comment: no link or reference for protocol. Report only results from central institute, not the tests performed locally for safety purposes.
7) Risk of confounding (prior exposure; other drugs/conditions; reporting of other steroids; baseline adrenal function)	High risk	P210: "corticosteroids allowed until one week before the study".<br>Comment: one week seems short but unclear (given lack of current info) if this would influence the numbers suppressed. Assume not on other steroids throughout.<br><br>Comment: patients with "clinically important hepatic, renal, cardiovascular, or psychiatric conditions" (p210) were excluded. Not on immunosuppressive drugs. This is not a blanket exclusion of any serious medical conditions, including cancer, or other medications.<br><br>Comment: 5-14% of patients had abnormal adrenal function at baseline.

3. Cydulka et al. 1998

Paper assessed:

Cydulka RK, Emerman CL. A pilot study of steroid therapy after emergency department treatment of acute asthma: is a taper needed? The Journal of Emergency Medicine 1998;16(1):15-9.

Entry	Judgement	Support for judgement
1) Random sequence generation (selection bias)	Low risk	P16 “computer-generated randomization Table”
2) Allocation concealment (selection bias)	Unclear risk	Comment: no details given
3) Blinding of participants and personnel (performance bias)	Low risk	P16 “Patients in the nontaper group were given eight 5-mg tablets to take each day. Patients in the taper group were given eight tablets to take each day: 5-mg prednisone tablets, up to the daily dose of prednisone, plus placebo look-alike tablets comprising the remainder of the eight tablets.”
4) Blinding of outcome assessment (reporting bias)	Low risk	Comment: no details; ACTH test unlikely to be influenced by knowledge of allocation.
5) Incomplete outcome reporting (attrition bias)	Low risk	Comment: no loss to follow-up reported
6) Selective reporting (reporting bias)	Unclear risk	Comment: no link or reference for protocol.
7) Risk of confounding (prior exposure; other drugs/conditions; reporting of other steroids; baseline adrenal function)	Unclear risk	P16: "already using inhaled or oral steroids, those requiring chronic steroid use, as defined by daily steroid use, or those who had required steroids within 2 weeks of admission to the ED were also excluded".<br>Comment: no inhaled/oral steroids for at least 2 weeks; no mention of other routes – assume excluded.<br><br>Comment: excluded patients with other lung conditions, diabetes or severe hypertension. This is not a blanket exclusion of other serious medical conditions. No mention of other therapies.<br><br>Comment: no adrenal suppression at presentation (table 1).

4. Feiss et al. 1992

Paper assessed:

Feiss G, Morris R, Rom D, Mansfield L, Dockhorn R, Ellis E, et al. A comparative study of the effects of intranasal triamcinolone acetonide aerosol (ITAA) and prednisone on adrenocortical function. The Journal of Allergy and Clinical Immunology 1992;89(6):1151-6.

**Entry**	**Judgement**	**Support for judgement**
1) Random sequence generation (selection bias)	Unclear risk	P1152 "subjects were randomly assigned to four groups"<br>Comment: no further details
2) Allocation concealment (selection bias)	Unclear risk	Comment: not discussed
3) Blinding of participants and personnel (performance bias)	Low risk	P1152: "The ITAA, oral placebo, aerosol placebo, and prednisone were supplied by Rorer Pharmaceutical…"<br><br>Comment: double blind – has placebo inhaler and tablets
4) Blinding of outcome assessment (reporting bias)	Low risk	Comment: no details; ACTH test unlikely to be influenced by knowledge of allocation.
5) Incomplete outcome reporting (attrition bias)	Low risk	Comment: For whole study, 2 of 64 patients were lost to follow-up. Infer from table 1 one each lost from inhaled groups.
6) Selective reporting (reporting bias)	Unclear risk	Comment: no link or reference for protocol.
7) Risk of confounding (prior exposure; other drugs/conditions; reporting of other steroids; baseline adrenal function)	Low risk	P1152 "None of these subjects had a history of treatment with any glucocorticoid within the previous year."<br>P1152 "no concomitant glucocorticoid preparations were permitted during the treatment period."<br>Comment: no recent glucocorticoids.<br><br>P1152: "subjects were otherwise healthy without conditions or concomitant medications that could have altered glucocorticoid disposition."<br><br>P1152: "Before treatment, all subjects had normal stimulation responses"

5. Ferguson et al. 1998

Paper assessed:

Ferguson A, Campieri M, Doe W, Persson T, Nygard G. Oral budesonide as maintenance therapy in Crohn's disease--results of a 12-month study. Global Budesonide Study Group. Alimentary Pharmacology & Therapeutics 1998;12(2):175-83.

Entry	Judgement	Support for judgement
1) Random sequence generation (selection bias)	Low risk	p 176 "Patients were randomly allocated to either budesonide CIR, 3mg or 6 mg daily, or placebo" … "Randomization was made in blocks of 6 and opaque assignment envelopes were sequentially numbered and sealed."<br>Comment: assume done
2) Allocation concealment (selection bias)	Low risk	p 176 "opaque assignment envelopes were sequentially numbered and sealed."<br>Comment: assume done
3) Blinding of participants and personnel (performance bias)	Low risk	P176 "Placebo capsules, of an identical appearance to the budesonide CIR 3 mg capsules"<br>P176 "The study was blinded using a double-dummy technique, and active drug and placebo were packed in blister packs"<br>Comment: assume done
4) Blinding of outcome assessment (reporting bias)	Low risk	Comment: no details; ACTH test unlikely to be influenced by knowledge of allocation.
5) Incomplete outcome reporting (attrition bias)	Unclear risk	Comment: total of 75 patients randomised. All patients followed up (Table 2). Large proportion in each group discontinued prematurely – unclear the % at 12 weeks. No ACTH results for 6/22 and 7/26 of the patients, with no explanation for why they were untested.
6) Selective reporting (reporting bias)	Unclear risk	Comment: no link or reference for protocol.
7) Risk of confounding (prior exposure; other drugs/conditions; reporting of other steroids; baseline adrenal function)	High risk	Comment: all patients recruited straight from previous study, exposed to 9mg budesonide or 40mg prednisolone; know that some of these patients had AI after first study.<br><br>Comment: for initial study (Campieri et al. 1997) patients with "clinically important hepatic, renal, cardiovascular, or psychiatric conditions" (p210) were excluded. Not on immunosuppressive drugs. This is not a blanket exclusion of any serious medical conditions, including cancer, or other medications.

6. Greenberg et al. 1996

Paper assessed:

Greenberg GR, Feagan BG, Martin F, Sutherland LR, Thomson AB, Williams CN, et al. Oral budesonide as maintenance treatment for Crohn's disease: a placebo-controlled, dose-ranging study. Canadian Inflammatory Bowel Disease Study Group. Gastroenterology 1996;110(1):45-51.

**Entry**	**Judgement**	**Support for judgement**
1) Random sequence generation (selection bias)	Low risk	P46 "Randomization was by computer-generated lists and was stratified by centre."
2) Allocation concealment (selection bias)	Unclear risk	Comment: no details given
3) Blinding of participants and personnel (performance bias)	Low risk	P46 "The placebo was identical in appearance to the investigational drug"
4) Blinding of outcome assessment (reporting bias)	Low risk	Comment: no details; ACTH test unlikely to be influenced by knowledge of allocation.
5) Incomplete outcome reporting (attrition bias)	Unclear risk	Comment: 105 patients were randomised. 74 did not complete the study (52 weeks). 2 were lost to follow-up. Unclear if all patients were tested (13 weeks). Discontinuation/relapse earlier in lower dose group.
6) Selective reporting (reporting bias)	Unclear risk	Comment: no link or reference for protocol.
7) Risk of confounding (prior exposure; other drugs/conditions; reporting of other steroids; baseline adrenal function)	High risk	Comment: recruited from previous 8 week trail, exposed to 0, 3, 9 or 15mg of budesonide. 44-36% of patients had abnormal baseline ACTH results.<br><br>Comment: excluded patients with diabetes, infection, peptic ulcer disease, cancer, clinically important cardiac of hepatic disease; no mention of other HPA conditions. No mention of other therapies (unclear risk).

7. Hellers et al. 1999

Paper assessed:

Hellers G, Cortot A, Jewell D, Leijonmarck CE, Lofberg R, Malchow H, et al. Oral budesonide for prevention of postsurgical recurrence in Crohn's disease. The IOIBD Budesonide Study Group. Gastroenterology 1999;116(2):294-300.

Entry	Judgement	Support for judgement
1) Random sequence generation (selection bias)	Unclear risk	Comment: no details about the randomization technique
2) Allocation concealment (selection bias)	Low risk	P295 "The randomization code was not broken until each patient's file was complete and approved for statistical analysis and adverse event evaluation"<br><br>Comment: implies unaware until then, but no further details.
3) Blinding of participants and personnel (performance bias)	Low risk	Comment: no details other than 'double blind' – probably done
4) Blinding of outcome assessment (reporting bias)	Low risk	P295 "The randomization code was not broken until each patient's file was complete and approved for statistical analysis and adverse event evaluation" ACTH test unlikely to be influenced by knowledge of allocation.
5) Incomplete outcome reporting (attrition bias)	Unclear risk	Comment: 130 patients were randomised. 1 patient (treated group) was lost to follow-up. 40/63 treated completed study. Only proportions given, unclear whether all patients were tested.
6) Selective reporting (reporting bias)	Unclear risk	Comment: no link or reference for protocol.
7) Risk of confounding (prior exposure; other drugs/conditions; reporting of other steroids; baseline adrenal function)	High risk	Comment: no systemic glucocorticoids for at least 30 days. No concurrent Crohn's disease medication, limited info about other medication. No information about other medication conditions.<br><br>Comment: 33% of patients in the treated group had abnormal adrenal function at baseline.

8. Kirwan et al. 2006

Paper assessed:

[1] Kirwan JR, Hickey SH, Hallgren R, Mielants H, Bjorck E, Persson T, et al. The effect of therapeutic glucocorticoids on the adrenal response in a randomized controlled trial in patients with rheumatoid arthritis. Arthritis and Rheumatism 2006;54(5):1415-21.

Further details from:

[2] Kirwan JR, Hällgren R, Mielants H, Wollheim F, Bjorck E, Perrson T, et al. A randomised placebo controlled 12 week trial of budesonide and prednisolone in rheumatoid arthritis. Annals of the Rheumatic Diseases 2004;63(6):688-95

Entry	Judgement	Support for judgement
1) Random sequence generation (selection bias)	Low risk	P689[2]: "Drugs were dispensed by the hospital pharmacy in relation to the study number and in accordance with a predefined sequence of randomly generated allocations ….”
2) Allocation concealment (selection bias)	Low risk	P689[2]: "… predefined sequence of randomly generated allocations kept in sealed envelopes.”
3) Blinding of participants and personnel (performance bias)	Low risk	P690[2]: “One preparation was either budesonide 9 mg, budesonide 3 mg, or budesonide placebo, all of which were prepared as identical capsules. The other two preparations were either prednisolone 5 mg plus prednisolone 2.5 mg, or identical prednisolone 5 mg placebo and 2.5 mg placebo tablets.”<br><br>Comment: double dummy – the budesonide and bud placebo are identical, as are the prednisolone and pred placebo
4) Blinding of outcome assessment (reporting bias)	Low risk	P689[2]: “The double blind nature of the study was maintained until after all patients had completed the follow up period.”<br><br>P1416[1]: “Adrenal function<br>was measured at baseline and after 12 weeks, using the short ACTH stimulation test”<br><br>Comment: ACTH test unlikely to be influenced by knowledge of allocation.
5) Incomplete outcome reporting (attrition bias)	Unclear risk	Comment: 143 patients were randomised. One had no study medication and was not included in the analysis. 139/142 had ACTH test at start, 134/142 at end. (max ~8% (3/35) per group missing – Table 1[1])<br><br>Comment: unclear why they were not tested.
6) Selective reporting (reporting bias)	Unclear risk	Comment: no link or reference for protocol.
7) Risk of confounding (prior exposure; other	High risk	P1416[1]: "no glucocorticoids had been given by any route for at least 30 days".

drugs/conditions; reporting of other steroids; baseline adrenal function)		P689[2]: "patients were excluded from the study if … other significant disease as judged by the investigator"<br>Comment: list of diseases explicitly excluded given in [2]. This does not include e.g. cancer or HPA dysfunction, but would probably be covered by the above statement. Therapies other than RA treatments are not mentioned.<br><br>Comment: a small number of patients (up to 3 which could be up to 8%) per group had abnormal plasma cortisol at baseline.

9. Lofberg et al. 1996

Paper assessed:

Lofberg R, Rutgeerts P, Malchow H, Lamers C, Danielsson A, Olaison G, et al. Budesonide prolongs time to relapse in ileal and ileocaecal Crohn's disease. A placebo controlled one year study. Gut 1996;39(1):82-6.

Entry	Judgement	Support for judgement
1) Random sequence generation (selection bias)	Low risk	P83 "The patients were randomised at each centre in blocks of six"
2) Allocation concealment (selection bias)	Unclear risk	Comment: no details given
3) Blinding of participants and personnel (performance bias)	Low risk	P83 "Each capsule contained 3 mg of budesonide. Placebo capsules of identical appearance were manufactured by Astra Draco."
4) Blinding of outcome assessment (reporting bias)	Low risk	P83 "All plasma samples were analysed in a blinded manner at Astra Draco, Lund using a high performance liquid chromatography method."
5) Incomplete outcome reporting (attrition bias)	High risk	Comment: 90 patients randomised. No loss to follow-up. ACTH results shown for 23/32, 21/31 and 13/27 at three months. Reason for missing not given – potentially due to therapeutic failure.
6) Selective reporting (reporting bias)	Unclear risk	Comment: no link or reference for protocol.
7) Risk of confounding (prior exposure; other drugs/conditions; reporting of other steroids; baseline adrenal function)	High risk	P82: "Patients entering the trial had been treated during 10 weeks with either budesonide or prednisolone for active CD in a preceding study".<br><br>Comment: some conditions explicitly excluded (diabetes, cardiovascular) however incomplete list. Some treatments were excluded (concurrent CD medication, other corticosteroids, immunosuppressants etc.).<br><br>P84: "Forty two per cent of the patients in the 6mg budesonide group had a plasma cortisol value below the lower reference value. …"

10. Rutgeerts et al. 1994

Paper assessed:

Rutgeerts P, Lofberg R, Malchow H, Lamers C, Olaison G, Jewell D, et al. A comparison of budesonide with prednisolone for active Crohn's disease. The New England journal of medicine 1994;331(13):842-5.

Entry	Judgement	Support for judgement
1) Random sequence generation (selection bias)	Low risk	P843: "There was separate randomization of patients in blocks of four at each centre"
2) Allocation concealment (selection bias)	Unclear risk	Comment: no details given
3) Blinding of participants and personnel (performance bias)	Low risk	P843: "All patients received budesonide capsules and prednisolone tablets simultaneously, but in each case on of the pills was an identical-appearing placebo".
4) Blinding of outcome assessment (reporting bias)	Low risk	Comment: no details; ACTH test unlikely to be influenced by knowledge of allocation.
5) Incomplete outcome reporting (attrition bias)	Unclear risk	Comment: 176 patients were randomised. Two patients withdrew before week 2 and are not included. 16/88 and 15/88 withdrew overall. Is not possible to see the numbers tested throughout.
6) Selective reporting (reporting bias)	Unclear risk	Comment: no link or reference for protocol.
7) Risk of confounding (prior exposure; other drugs/conditions; reporting of other steroids; baseline adrenal function)	Unclear risk	Comment: no corticosteroids in preceding 2 weeks. List of conditions excluded specified, incomplete. No information about excluded therapies.<br><br>Comment: baseline levels of cortisol are not given.

11. Sorkness et al. 1999

Paper assessed:

Sorkness CA, LaForce C, Storms W, Lincourt WR, Edwards L, Rogenes PR. Effects of the inhaled corticosteroids fluticasone propionate, triamcinolone acetonide, and flunisolide and oral prednisone on the hypothalamic-pituitary-adrenal axis in adult patients with asthma. Clinical Therapeutics 1999;21(2):353-67.

Entry	Judgement	Support for judgement
1) Random sequence generation (selection bias)	Low risk	p355 "multicentre, randomised placebo-controlled, double-masked…. …parallel-group studies"<br>p357 "Computer-generated randomization resulted in comparable treatment groups at baseline…"
2) Allocation concealment (selection bias)	Unclear risk	Comment: no mention of allocation.
3) Blinding of participants and personnel (performance bias)	Low risk	p355 "In study 1, prednisone (10 mg) and a placebo capsule were taken once daily in the morning. " "Placebo inhales were manufactured to ensure a double-masked double- or triple-dummy design".
4) Blinding of outcome assessment (reporting bias)	Low risk	Comment: no details; ACTH test unlikely to be influenced by knowledge of allocation.
5) Incomplete outcome reporting (attrition bias)	High risk	Comment: 168 patients enrolled. 4 patients were withdrawn. 1/28 patients (4%) in prednisolone group was untested. Withdrawals were due to adverse events.
6) Selective reporting (reporting bias)	Unclear risk	Comment: no link or reference for protocol.
7) Risk of confounding (prior exposure; other drugs/conditions; reporting of other steroids; baseline adrenal function)	Low risk	P355: "To be eligible for study participation, patients were required to have a baseline prestimulation morning plasma cortisol >=7mcg/dl…" (etc.)<br><br>Comment: excluded if any corticosteroid or immunosuppressive in prior 3 months or use of any therapy known to cause abnormal response to exogenous GCs. No mention of other medical conditions.

12. Suzuki et al. 2013

Paper assessed:

Suzuki Y, Motoya S, Takazoe M, Kosaka T, Date M, Nii M, et al. Efficacy and tolerability of oral budesonide in Japanese patients with active Crohn's disease: a multicentre, double-blind, randomized, parallel-group Phase II study. Journal of Crohns & Colitis 2013;7(3):239-47.

Entry	Judgement	Support for judgement
1) Random sequence generation (selection bias)	Low risk	P240 “After a 3-week screening period, eligible patients were randomized (using a validated computer program provided by the study sponsor) to one of three oral once-daily (od) treatment groups”
2) Allocation concealment (selection bias)	Unclear risk	Comment: not discussed
3) Blinding of participants and personnel (performance bias)	Low risk	P240 “To maintain blinding, all patients took 5 matching capsules od before breakfast, with each capsule containing either budesonide 3 mg or placebo. At treatment end (or following early discontinuation) patients underwent a 2-week tapering period, during which blinding was maintained”
4) Blinding of outcome assessment (reporting bias)	Low risk	Comment: no details; ACTH test unlikely to be influenced by knowledge of allocation.
5) Incomplete outcome reporting (attrition bias)	High risk	Comment: 77 patients were randomised. 14 patients withdrew. 16/26, 18/25 and 13/26 patients have ACTH results. This is lower than the numbers completing the study. No reasons given for those missing results. No patients withdrew due to GC-related AE.
6) Selective reporting (reporting bias)	Unclear risk	Comment: link to record on clinicaltrials.gov. HPA testing not mentioned as an outcome - remains unclear whether this was the only test used.
7) Risk of confounding (prior exposure; other drugs/conditions; reporting of other steroids; baseline adrenal function)	High risk	P240: "Patients were excluded if they … had a serious concomitant disease or any clinically relevant abnormal laboratory results at baseline.<br><br>Comment: patients taking GCs excluded (time-frame unclear)<br><br>Comment: up to 44% of patients had abnormal ACTH result at baseline.

13. Vargas et al. 1998

Paper assessed:

Vargas R, Dockhorn RJ, Findlay SR, Korenblat PE, Field EA, Kral KM. Effect of fluticasone propionate aqueous nasal spray versus oral prednisone on the hypothalamic-pituitary-adrenal axis. The Journal of Allergy and Clinical Immunology 1998;102(2):191-7.

Entry	Judgement	Support for judgement
1) Random sequence generation (selection bias)	Unclear risk	P192: "randomised, double-blind…"<br>Comment: no details given
2) Allocation concealment (selection bias)	Unclear risk	Comment: no details given
3) Blinding of participants and personnel (performance bias)	Low risk	P193 "For double-blinding purposes, tablets containing 2.5 mg of prednisone (Deltasone; The Upjohn Co., Kalamazoo, Mich.) were encapsulated, and bioequivalence of encapsulated and nonencapsulated prednisone was confirmed before study initiation. Each patient took both a morning and evening nasal spray (active or placebo) and two capsules in the morning (active or placebo)."
4) Blinding of outcome assessment (reporting bias)	Low risk	Comment: no details; ACTH test unlikely to be influenced by knowledge of allocation.
5) Incomplete outcome reporting (attrition bias)	Unclear risk	Comment: 105 patients randomised (Table 1). Measurements are missing for 3-5 patients with no explanations.
6) Selective reporting (reporting bias)	Unclear risk	Comment: no link or reference for protocol; present results such that can only use one of the response criteria, cannot combine them.
7) Risk of confounding (prior exposure; other drugs/conditions; reporting of other steroids; baseline adrenal function)	Low risk	Comment: participants were required to have normal adrenal function at baseline.<br><br>Comment: concomitant medications which could alter adrenal function, including local (2 months) and systemic (3 months) steroids were excluded.<br><br>Comment: conditions known to cause abnormal response to exogenous steroids or to affect HPA axis assessments were excluded.

References

1 Aaronson D, Kaiser H, Dockhorn R, Findlay S, Korenblat P, Thorsson L, et al. Effects of budesonide by means of the Turbuhaler on the hypothalmic-pituitary-adrenal axis in asthmatic subjects: a dose-response study. The Journal of Allergy and Alinical Immunology 1998;101(3):312-9.

2 Campieri M, Ferguson A, Doe W, Persson T, Nilsson LG. Oral budesonide is as effective as oral prednisolone in active Crohn's disease. The Global Budesonide Study Group. Gut 1997;41(2):209-14.

3 Cydulka RK, Emerman CL. A pilot study of steroid therapy after emergency department treatment of acute asthma: is a taper needed? The Journal of Emergency Medicine 1998;16(1):15-9.

4 Feiss G, Morris R, Rom D, Mansfield L, Dockhorn R, Ellis E, et al. A comparative study of the effects of intranasal triamcinolone acetonide aerosol (ITAA) and prednisone on adrenocortical function. The Journal of Allergy and Clinical Immunology 1992;89(6):1151-6.

5 Ferguson A, Campieri M, Doe W, Persson T, Nygard G. Oral budesonide as maintenance therapy in Crohn's disease--results of a 12-month study. Global Budesonide Study Group. Alimentary Pharmacology & Therapeutics 1998;12(2):175-83.

6 Greenberg GR, Feagan BG, Martin F, Sutherland LR, Thomson AB, Williams CN, et al. Oral budesonide as maintenance treatment for Crohn's disease: a placebo-controlled, dose-ranging study. Canadian Inflammatory Bowel Disease Study Group. Gastroenterology 1996;110(1):45-51.

7 Hellers G, Cortot A, Jewell D, Leijonmarck CE, Lofberg R, Malchow H, et al. Oral budesonide for prevention of postsurgical recurrence in Crohn's disease. The IOIBD Budesonide Study Group. Gastroenterology 1999;116(2):294-300.

8 Kirwan JR, Hickey SH, Hallgren R, Mielants H, Bjorck E, Persson T, et al. The effect of therapeutic glucocorticoids on the adrenal response in a randomized controlled trial in patients with rheumatoid arthritis. Arthritis and Rheumatism 2006;54(5):1415-21.

9 Lofberg R, Rutgeerts P, Malchow H, Lamers C, Danielsson A, Olaison G, et al. Budesonide prolongs time to relapse in ileal and ileocaecal Crohn's disease. A placebo controlled one year study. Gut 1996;39(1):82-6.

10 Rutgeerts P, Lofberg R, Malchow H, Lamers C, Olaison G, Jewell D, et al. A comparison of budesonide with prednisolone for active Crohn's disease. The New England journal of medicine 1994;331(13):842-5.

11 Sorkness CA, LaForce C, Storms W, Lincourt WR, Edwards L, Rogenes PR. Effects of the inhaled corticosteroids fluticasone propionate, triamcinolone acetonide, and flunisolide and oral prednisone on the hypothalamic-pituitary-adrenal axis in adult patients with asthma. Clinical Therapeutics 1999;21(2):353-67.

12 Suzuki Y, Motoya S, Takazoe M, Kosaka T, Date M, Nii M, et al. Efficacy and tolerability of oral budesonide in Japanese patients with active Crohn's disease: a multicentre, double-blind, randomized, parallel-group Phase II study. Journal of Crohns & Colitis 2013;7(3):239-47.

13 Vargas R, Dockhorn RJ, Findlay SR, Korenblat PE, Field EA, Kral KM. Effect of fluticasone propionate aqueous nasal spray versus oral prednisone on the hypothalamic-pituitary-adrenal axis. The Journal of Allergy and Clinical Immunology 1998;102(2):191-7.
