# Supplementary material for: Systemic glucocorticoid therapy and adrenal insufficiency in adults: A systematic review
Source: Semin Arthritis Rheum. 2016 Aug;46(1):133–41. doi: 10.1016/j.semarthrit.2016.03.001 (PMC4987145; doi:10.1016/j.semarthrit.2016.03.001)

Supplementary File 5 Group size vs. %AI (Risk of bias across studies)

(The y-axis is truncated at 150, excluding two groups. One had n=279, %AI=63.1, the other n=399, %AI=13.5)

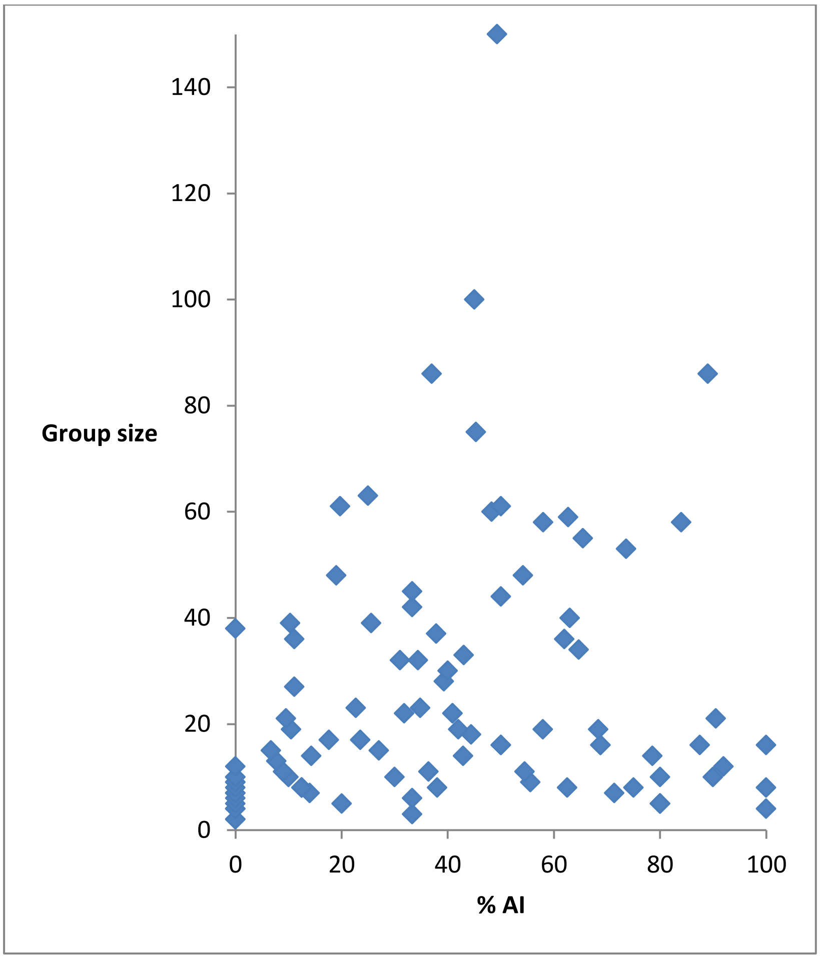

Supplement: Supplementary file 5 — Supplementary material [file mmc5.pdf]
